# On the Deserts and Duties of the Physician

**Published:** 1846-07

**Authors:** 


### ART. X.

*Sui Pregi e Doveri del Medico.* Del Professore ROBERTO SAVA, &c. &c.—*Milano,* 1845.

*On the Deserts and Duties of the Physician.* By Professor ROBERT SAVA.—*Milan,* 1845. 8vo, pp. 214.

WE have gone through the work before us with considerable interest, as it is calculated to afford information upon the condition and sentiments of our professional brethren in Italy, a subject well deserving attentive examination, and one which the standing and position of the author have enabled him to treat satisfactorily. The work is divided into a number of chapters, and we think we cannot do better than follow the author's arrangement, extracting the parts most worthy of notice, and making our own comments upon them.

The work is commenced by a short introduction on the rank of the profession, which the following extract will prove that Dr. Sava is not at all disposed to depreciate: it is thoroughly Italian:

"A great physician is the *first of men:* by the improvements with which he perfects the art of healing he becomes the benefactor of humanity; and by the empire he exercises over death he is in some degree the image of the divinity upon earth." (p. 11.)

It is stated a little further on that Molière by his sarcasms has done a great service to the profession, by correcting the defects of physicians, and rendering them, "what in reality they now are, the most intelligent and agreeable men in society," and that all agree in treating them as such. If this be true with regard to Italy, we can cordially congratulate our brethren on the position they have acquired; but some personal observation and many remarks of the author in other chapters lead us to fear that "the wish was father to the thought."

The first chapter is on the "preparatory studies of persons destined for the profession, and on their conduct at college," insisting on selecting such youths only for a medical career who have a natural disposition for it, giving them a sound general education, and then pointing out much such a course of study as is adopted in our medical schools.

The "entrance of a young physician into the world" is the subject of the second chapter. It appears that many of the evils recognized here are in full operation in Italy—the same boldness and confidence of ignorance, the same poverty tempting to charlatanry, the same supposed neglect of patient merit. Let us hear our author:

"The qualities essential to the physician to succeed in the world are not transcendent merit, great devotion to study, and profound judgment; but rather an exorbitant accumulation of charlatanry, untiring chattering, and an audacity that nothing can ever disconcert." (p. 29.)

Not a very flattering portrait of the "first of men!"

We pass over an unimportant chapter on the "estimation of the physician," and arrive at the fourth, on the "physician in society." To prevent repetition we shall connect our remarks on this chapter with others on the ninth and sixteenth, "the exterior of the physician," and "*saper-fare,*" or knowledge of the world. The author commences with some just observations:

"Medicine is not a science incompatible with indulgence in social amusement, nor does it exclude those who exercise it from the politeness, amenity, and graces that form the pleasure of society. One may be at the same time physician and man of the world; and, if some melancholy doctors declaim against the study of the art of pleasing, in doing so they have less regard to the dignity of their profession than to the impossibility of correcting the pedantry of their character and the absurdity of their manners. The impertubrable gravity they carry into society, as well as to the bedside, is a veil under which they frequently conceal profound ignorance; and the silly sarcasms they launch against those of their colleagues who join a clear intellect and agreeable manners to knowledge, are nothing more than a confession of their secret jealousy. There are close connexions between the arts of pleasing and healing....... Some young physicians, too much engaged by study, live with their books, and withdraw themselves from society to dedicate themselves to their learned researches. This constant occupation gives them an embarrassed aspect and a timid reserve, of which they can never correct themselves, and which frequently diminish the success to which the multiplicity and profundity of their acquirements entitle them." (pp. 38-40.)

Perhaps the young medical man, in his entrance into the world, is never so likely to err as in taking the happy medium between pedantry on the one hand, and fashionable frivolity on the other; he is apt to run into either of these extremes, so injurious to his prospects. There is a powerful passage in Lord Brougham's dissertation upon the character of Sir Humphrey Davy we cannot forbear quoting. He says, "He was led away by the plaudits of fashion, and must needs join in its frothy, feeble current. For a while he is remarked to have shown the incongruous combination of science and fashion, which form a most imperfect union, and produce a compound of no valuable qualities, somewhat resembling the nitrous gas on which he experimented earlier in life, having an intoxicating effect on the party tasting it, and a ludicrous one on all beholders." Still *omnibus ornatum excellere rebus,* should be the motto of the medical man striving for excellence; for if sound solid learning be not accompanied by the knowledge of a man of the world, and the habits and manners of a gentleman, it will become tainted with pedantry, and its use be confined principally to the closet; and thus be far less likely to enhance the success of its possessor. The social pleasures of the evening need never interfere with the business and study of the day, and, enjoyed in moderation, are useful in affording relaxation to the mind, and in forming manners which give a lustre to knowledge, adorn it, and prepare a path for its progress. Lord Chesterfield, in his Letters to his Son, has observed, with great truth, that "the exercise of the mind in the morning whets the appetite for the pleasures of the evening, as much as the exercise of the body whets the appetite for dinner. Business and pleasure, rightly understood, mutually assist each other, instead of being enemies, as silly or dull people often think them. No man tastes pleasures truly who does not earn them by previous business; and *few people do business well who do nothing else.*" Most people must have seen genuine merit obscured by the want of a little of the polish required by refined social intercourse. Men of solid acquirements, in the presence of patients much their superiors in position, are sometimes, on this very account, awkward and disconcerted, uncertain what to do, what questions to put, or what replies to give; and either becoming disagreeably forward in their efforts to overcome their shyness, or so confused that they cannot readily apply their knowledge, and the confidence of the patient is lost. This is, no doubt, often the true source of the complaints we hear of ignorant and unworthy persons being high in public estimation, while merit is neglected, unrewarded, or oppressed. We believe that transcendant merit must naturally triumph over all obstacles; but unless it be of this commanding order, it will not serve in the choice between a nervous, timid, or ill-bred man, and his collected, easy, and agreeable, though perhaps less learned, rival.

In the chapter on the "exterior of the physician," the subject of dress is treated at some length; but the end of the author's arguments is simply that every pretension to singularity should be avoided—negligence and the extreme of fashion with equal care. Carelessness in dress is offensive to most persons, as it shows an apparent indifference to please, and thus becomes an indirect offence to their self-love; and slovenliness, especially in a young man, is too often connected with a want of that extreme cleanliness which health requires, and sometimes to a degree that shocks common decency.

In the chapter on "Saper-fare," the *savoir faire* of the French, for which our language has no exact equivalent, there is a curious passage, which we quote, as it may illustrate the position of the profession in Italy. The author is speaking of the method of making a name, and says,

"It is very difficult for a young physician to make himself known in a large and spacious city. Here are accumulated a prodigious quantity of doctors of every class: officers of public health, army surgeons, surgeons of the administration, obstetricians, titled physicians, physicians without title and without name, midwifes, &c. Here flourish charlatans of every species, from the herbalist, the homœopathist, the orthopedist, to the truss-maker and the curer of venereal diseases; even the druggists, with syringe or pestle in hand, mutilate prescriptions and give advice. What troubles, therefore, what labours, what circumspection are necessary to emerge from the crowd! How can the modest physician elevate alone the edifice of his renown? What time will be necessary to complete it?" (p. 113.)

We continue our extracts from this chapter, pointing out the trickery—which, though not actually recommended, is said to be necessary,—for marked reprobation, although it is to be feared that the progress of education is not so far advanced, even in our own country, as to render all that is said by any means untrue.

"The *saper-fare* of a physician may have for its object either glory or fortune. Few direct themselves towards the former; the crowd precipitates itself towards the latter. It is too difficult, even with great intrigue, to create a literary fame; but sure and ready means of becoming opulent offer themselves freely to the able and impudent man, who has prepared his success by subverting every sentiment of modesty and polite delicacy........ Some physicians, at the bedside of a patient to whom they wish to give a lofty idea of their knowledge, listen to him with great gravity, affect a profound comprehension, pronounce a few words in a most magisterial tone, and hurry away. Another oppresses the patient and those who surround him with questions, not to satisfy himself upon any obscure points of diagnosis, but to convey a deep impression of his exactitude and ability in the difficult art of observation. A third, previously instructed with regard to the etiology, symptoms, and nature of the disease by a servant, or friend, or the ordinary medical attendant, narrates to the patient, before questioning him, a faithful history of his sufferings; and all the bystanders, and the patient himself, surprised and ecstatic at such wonderful sagacity, are completely satisfied.........A physician of no great renown would positively compromise his interests if he limited himself to the prescription of simple remedies. The general ignorance obliges him to be a proselyte of the *polifarmacia*, so far as he can with slight deviation from reasonable rules; and, indeed, it is very easy to unite to any medicine a greater or less number of substances which are quite incapable of modifying its properties. And it is also useful frequently to vary the medicines, because the public readily doubts the knowledge of a physician who always prescribes the same remedies.

"Persons, even of the highest social rank, have such obstinate prejudices with regard to the properties of medicines, that it would be dangerous to contradict them. They admire, of all things, a multiplicity of remedies, and regard as extraordinary excellencies, the singularity of their names, their rarity, and, especially, their high price. Physicians, never prescribe to persons of this character those precious vegetables, well known and of daily use by the vulgar, which nature produces in abundance in the fields of our country; reserve these for the rabble. Do you wish to make a deep impression of your genius? Prescribe always extraordinary remedies, or rare substances brought at an exorbitant expense from the most distant countries." (pp. 115-17.)

There is some satire here not altogether undeserved; and equal, if not more, truth in the following:

"Success in practice contributes most materially to form the reputation of a physician. The principal means of obtaining this success is to limit one's self to a reasonable system of expectation, and to prescribe in cases in which no active medicine is clearly indicated, substances incapable of exciting remarkable changes in the animal economy. In the treatment of the greater number of internal diseases, regimen and hygienic means constantly suffice to restore health to its normal type. It has been demonstrated that in these cases, medicines advised by authors exasperate the symptoms, and frequently produce complications. A judicious physician, therefore, ought to observe the fundamental rule of practice—the frequent necessity of leaving nature to act." (p. 120.)

"Some great doctors, as a principle of *saper-fare*, maintain an aspect strangely preoccupied. Their appearance is that of a man immersed in profound meditation; their exterior is neglected, like that of a philosopher completely devoted to the study of important sciences; grave in their discourse, they express themselves only by laconic aphorisms; in the streets, the public squares, the most frequented places, indeed everywhere the multitude can see them, they affect distraction and reflection; there is a frown on the brow, the visage is lean, sallow, pallid, and black, with a rough beard; they dally along the streets, reading as they go some trifle, in order to appear full of practice, and unwilling to lose even that time to instruct themselves. But their hidden motive for pretending not to know the usages of society, or for not attending to its ceremonies, which they call trifles, is to give the appearance of being exclusively occupied by books and diseases, and their ardent desire to be brought forward as proof of the common axiom, that a little caprice or eccentricity is always connected with transcendant merit. At the bedside they listen very attentively, say a few words with measured gravity, take up their stick, and disappear. The quackery of these physicians appears in the midst of their affected doctorial gravity, like the pride of the Athenian through the holes of the torn mantle that covered him." (pp. 121-2.)

We continue to extract some of these amusing passages; they are not uninstructive. The author is speaking of the means of obtaining the confidence of patients:

"You are sent for by a lady, and hurry off immediately. The languid beauty, negligently reclined upon a sofa, opens an expiring eye, and with a hoarse, lamentable voice, commences the tremendous recital of sleeplessness which has tormented her the whole night; or draws an alarming picture of the agitation of her nerves, endowed with extreme irritability, as she says. Still the freshness of her full cheek shows nothing but the most perfect health. From your attentive examination, and the replies of the patient, you have already concluded that her sufferings are imaginary....... Oh, inconsiderate doctor! What! do you not see that she is determined to be ailing? Avoid such incautious imprudence—it would certainly ruin you, but listen with the most lively interest to the prolix history of the dreadful pains she says she suffers; diffuse the most affectionate advice and the most agreeable remedies; sympathize with that excessive susceptibility which subjects such attractions to continual anguish and repeated distress, and declaim against nature, which, according to woman all seducing beauty, all the graces and arts of pleasing, has diminished the value of such prerogatives, by giving her a too delicate organization—punishing her for being beautiful, by forming her too tender." (p. 125.)

Doubtless some artifice is not only allowable but useful in practice—a patient may be persuaded when he cannot be convinced, and influencing the imagination has great effect upon the body; but still, while we are disposed to allow with the author that very often the resources of art are uncertain and weak, and that the public is apt to attribute to the physician the insufficiency of medicine, we must most strongly object to the recommendation, not made in sarcasm in this place, "ordinarily to announce great danger or evil results from a disease, rather than a favorable termination or speedy convalescence." (p. 126.) This is as impolitic as cruel, and revolting to every better feeling of our nature. But we must pass to other topics.

We have a chapter on the "travels of a physician," which we only notice as containing some remarks on the Radcliffe Fellowship, while treating on the advantages resulting to medical science from supporting some physicians in foreign schools. Dr. Radcliffe left 600*l.* a year to two students of the University of Oxford, on condition that they should pass at least five years abroad; and our author remarks, we fear with too much truth, that carelessness in the selection of students, the nomination of whom rests with certain gentlemen, and the absolute want of regulations as to the employment of their time, have rendered useless a noble institution, which appeared to promise results of the greatest advantage.

In a chapter on "medical societies and medical journals," the following judicious passage is worthy of translation:

"An impartial journalist, in giving an account of a work, points out the errors and mistakes, but always respects the author, and never allows himself to launch against him any epigram. Bitter sarcasms nauseate, never persuade. He ought carefully to guard himself against the prejudices of enmity, and the prepossessions of friendship and esteem. These sometimes blind our aristarchi; at least, it is desirable that they should not oppress with the excess of their praises some persons not altogether without merit, but who are not in reality that which they wish to magnify them to be. Flattery injures the giver, without benefiting the receiver. Certain of our physicians are qualified as excellent writers, while it is plain from their works that they are ignorant of the elementary rules of the art of writing. ......Chénier has exactly defined the qualities that a good critic ough to possess. The ignorant person, he says, does not see what is beautiful; the detractor will not see it; the critic sees it, and puts it in evidence. He speaks of great authors that have passed away with respect, but not with idolatry. He is just to the dead, just and benevolent to the living. He does not limit himself to the admiration of grand works, but pays a tribute of esteem to useful labours. Criticism is the science of taste illuminated by justice...........The end of criticism is to instruct. To fulfil it a journalist should possess profound and varied knowledge, in order to judge soundly of the relations to the medical sciences. A vast erudition must be accompanied by the art of writing, and especially by good taste; without which his criticism would disgust his readers. And in giving an account of a new book, he should avoid every kind of digression, follow the path of the author, and bring forward his principal ideas, either to approve them, or compare them with others analogous, put forth by contemporaries or by the older writers—endeavouring always to vary his style according to the theme, in order to sustain the attention. The nature of the materials submitted to his criticism does not prevent him from writing with the necessary elegance." (pp. 48-53.)

We pass without further comment to a chapter on the "age of the physician." It is written to show that talent and not years is the true test of the practitioner; that knowledge makes a young man old, ignorance an old man a tyro.

In a following chapter on "literary habits in medical men," it is justly argued that he who devotes his whole time to the study of medicine does well, but that he who studies with equal ardour, and yet employs some time in general literature, does much better. The *belles-lettres* have the same effect upon the intellect that an excellent diet has upon the body. Haller and others are quoted as proofs that physicians may be good poets; but the author strongly dissuades them from publishing the verses they compose as a pastime. He thinks the ridicule attached to an indifferent poet compromises the dignity not only of the professor but of his art. The study of moral philosophy is recommended from its numerous relations with medicine. We thus trace the formation of ideas, the rules that ought to direct life, the paths to happiness, the influence that different climates exercise over the physical powers of man and on social institutions, the power of regimen upon the passions and intellectual faculties, and that of disease upon the operations of the intellect. We advance even to the operations that constitute the function of intelligence and determine the will, and learn to decipher the various characters of the passions. Nothing can ever separate the study of the physical man from the study of the moral man.

After a long chapter successfully combating the charge of atheism and irreligion brought against medical men in general, the author treats on the relations of physicians with each other. We are sorry to see it stated that "in general true friends are rarely found among individuals of the same profession; envy and interest are in opposition." (p. 98.) It says little for our Italian friends. The medical philosopher should be superior to the petty calculations of sordid interest; it is only base minds that envy affects. This the author fully allows, and urges the most scrupulous delicacy in all our dealings with each other. One of his hints will not be understood in Italy only.

"Nothing can be more shameful than the transactions which take place between some physicians and unscrupulous druggists. Every physician who feels the dignity of his profession will reject these illicit gains and vile associations." (p. 139.)

It is not every day that we can look over a series of portraits of the professional men of any nation, drawn by one of themselves; but here, in a chapter "on some defects which should be avoided in practice," there is quite a little picture gallery. We shall follow the guidance of Dr. Sava, and act as interpreter to our readers.

"1. *On routine, or mechanical practice.* The routine physician practises an art, the philosophical principles of which are unknown to him. Without good foundations, without medical genius, he reasons solely upon the perceptions of the senses. Aged in his ideas, indifferent to the progress of science, he obstinately confines himself within the limited circle of certain actions; and all his knowledge, all his ability, consist in seizing the first appearances of things, and in prescribing certain formulæ. He resembles the pilot in a tempestuous sea covered with rocks, who is ignorant of the existence of the compass as a guide in navigation. Very ignorant, therefore stubborn and obstinate, he is incapable of supporting the arduous labour and profound meditation that the difficult art of recognizing and curing diseases require from the practitioner. Like a machine, the wheels of which always determine the same effects, the routine physician always repeats the same acts. His indolent, dull, obtuse intellect is never applied to reflection, and abhors everything that has an appearance of labour: observation to him is dumb, and its brilliant light cannot clear away the dense cloud which closely covers his eyes.

"Many varieties of routine physicians may be distinguished—some, servile imitators of the ancients, are far from supposing that twenty centuries of experience have wrought any progress in medicine. Hippocrates was a great physician, yet he was not acquainted with tartar emetic and quinine—therefore in their opinion quinine and tartar emetic are useless remedies. Such physicians declaim against vaccination, and in general against all the discoveries of genius. Others unite to the most profound ignorance—to the absolute incapacity of appreciating the merit of the ancients, a stupid pride which will not allow them to recognize anything valuable in their contemporaries. No principle guides them, and they might paint themselves approaching blindfold the bedside of a patient, close to which death stands watchful and prompt, raising an axe impelled by chance . . . . .

"It is difficult to visit a great number of patients and defend one'sself from the tendency to blind medication induced in man by the natural slothfulness of his intellect, and on this account routine physicians especially abound in hospitals.

"These persons recognize a disease at a single glance—that which presents the most difficult diagnosis is characterized most easily by them—nothing embarrasses them. After a few questions made for form's sake to the patient, they mechanically prescribe a formula, which the student, after having heard the indicating word, writes at full length in the visiting-book. This is all their art—this is their conduct, always the same. But these practitioners, the number of whom is fortunately inconsiderable, scarcely know the faces of their patients.

"Some physicians become machines as they grow old; age does not allow them to follow the progress of science, or subject themselves to new studies: obstinately attached to their old doctrines, they will vary nothing; everything new they treat with disgust and disdain, and thus they never read. After fifty years of medical practice it is impossible for them to adopt other principles, different from those they have acquired, and been accustomed so long to follow.

"2. *On presumption.* Do not ask this doctor what he knows, but rather what he is ignorant of. He has read everything, has seen everything; the most delicate surgical operations to him are mere pastime; nothing confounds him: his genius foresees everything, undertakes everything. He speaks in magnificent periods, and considers it dishonorable to appear ignorant of anything. What diseases has he not cured? Confirmed cancer and hydrophobia in his hands have ceased to be incurable; without vanity he believes himself to possess all the knowledge that he can have, or can even acquire: the first aphorism of Hippocrates has no signification for him: in him he believes that the power and genius of Esculapius self are infused.

"3. *On timidity.* This physician has great talents, profound and vast knowledge, yet he does not succeed, and will never occupy a part in the hierarchy of his profession; and with the most extensive acquirements, he has the appearance of ignorance. Question him—his replies are most confused and inexact. The most simple cases alarm him—he detests acting, and always does it with fear.* In vain nature announces a favorable result—still trembling he dares not second her. He has never felt those sudden unlooked-for inspirations which reveal to the man of genius the character of a disease complicated in its progress and diagnosis, and make him discover in untrodden paths the means of overcoming its violence and obstinate resistance. Thus he loses in deliberation the favorable opportunity and the moment of risking with advantage. Such a physician does not kill his patients, but he lets them die.

"4. *On false judgment.* Some physicians puff themselves up with pride, boasting scepticism in their science. Free from every prejudice, they regard the precepts of the oracle of Cos as idle nonsense. Immoveable in their opinions, they look upon the most authentic facts as fables, and the art of knowing and treating disease is, in their eyes, quackery founded upon the ignorance and credulity of the vulgar. But how are we not imposed upon by persons initiated into all the secrets of medicine? How suspect them of unfairness, when in truth they make the sacrifice of so many years of study of such painful toil? In this manner many ignorant persons argue. Still the impartial man soon discovers in these pyrrhonians, these empirics, that, disgusted by an unfortunate practice, they impudently accuse medicine of the errors exclusively imputable to their own ignorance. Without learning or talent, ignorant of the elementary principles of science, with judgment essentially false, in order to appear freethinkers in the profession, they slander that of which they are ignorant, condemn everything they are incapable of comprehending, and become a mark of the public contempt for daring to exercise a calling they themselves condemn as useless to society.

"Other physicians perceive nothing obscure in the science of humanity. Nature has no secret they cannot discover—no veil hides from their penetrating regard the mysteries of our organization. There are no diseases they cannot perfectly explain and cure. They regulate themselves blindly by all the observations contained in books, and all the axioms of Hippocrates appear to them immutable truths. Experience would accuse their doctrine in vain—the teacher has said it,—they reply in his defence that he is never deceived. Thus for them all new discoveries in science are valueless, indeed they will not suppose them true. All the phenomena, all the changes that a disease presents during its course, depend in their eyes, not upon the efforts of nature, but upon the drugs they have administered, however inactive and useless these may have been, and with their lofty idea of the powers of medicine. They imagine that none of the evils which

* We once heard an old and experienced physician of this stamp declare, that though he had practised medicine fifty years, he had scarcely ever left a patient's house without feeling a desire to go back and alter his prescription! And yet our friend was a man of great intellectual power and much learning. He was only singularly deficient in force of character, or, as the phrenologists would say, in *combativeness*.—ED.

afflict the human species can resist them; and indiscriminate dispensers of tonics, bloodletting, emetics, and the more active remedies, they still think that they must act, and act with all energy.

"These are fanatics in medicine: such are the enthusiastic partisans of any doctrine. Let every one beware of censuring their venerated idol; if he should have such temerity, the abuse vomited from their mouth would equal in quantity the words that Homer makes the aged Nestor deliver in public discourse, comparing them to sheets of snow impetuously falling. They are filled by exclusive admiration, and despise even what is judicious in other schools." (pp. 140-7.)

We have given this chapter at length as a good specimen of the author's style—perhaps the portrait painting is not altogether free from caricature, but if it be true that men are more readily laughed out of their errors than preached out of them, it may not be without its good effect. The succeeding chapters "on the mode of questioning patients," "on the patience, prudence, charity, discretion, chastity, philanthropy, and magnanimity of the physician," "on the peculiar qualities of the surgeon," "on the duties of physicians to the dying," "on mental medicine," and "on fees," are sound and sensible, but rather too commonplace to be adapted to our pages. That on mental medicine is perhaps the worst in the book, being merely a superficial glance at a most important and interesting subject. We felt almost tempted to preach a sermon on the author's text, but our space is already exceeded, and we must close with thanks to Dr. Sava for a well-written and interesting book, full of sound, sensible, and acute observation. The only fault we find with it, is that self-love and self-interest are too much worked upon as main-springs of conduct, to the neglect of those high feelings of honour which should guide every one in his course through life.

On looking over some of our remarks on the manners of the physician, it seems to us possible that some of our readers may be led to believe that we attach undue importance to superficial accomplishments. We wish, therefore, to observe here, that, while we consider a deep and thorough knowledge of his profession to be the real claim of every medical man to distinction and reward, we are desirous that these claims should not be obscured by inattention to minor matters. The grand difference between the well-bred and ill-bred man, lies in the degree of attention paid to the feelings of others. A man of common sense, with a moderate supply of good nature, just sufficient to make himself cheerfully undergo a little self-denial for the sake of others, cannot be guilty of any real offence to good breeding. It requires a certain amount of observation and knowledge of the world to acquire unembarrassed, easy, and graceful manners; but the more the power of such manners is insisted upon in covering the want of merit, the more clearly do we see their influence in adorning it.

